# Characterizing the microbiome of ectoparasitic louse flies feeding on migratory raptors

**DOI:** 10.1371/journal.pone.0234050

**Published:** 2020-06-04

**Authors:** Rebecca A. McCabe, Joseph P. Receveur, Jennifer L. Houtz, Kayli L. Thomas, M. Eric Benbow, Jennifer L. Pechal, John R. Wallace

**Affiliations:** 1 Department of Natural Resource Sciences, McGill University, Sainte-Anne-de-Bellevue, Québec, Canada; 2 Department of Entomology, Michigan State University, East Lansing, Michigan, United States of America; 3 Department of Biology, Millersville University, Millersville, Pennsylvania, United States of America; University of Pretoria, SOUTH AFRICA

## Abstract

Louse flies (Diptera: Hippoboscidae) are obligate ectoparasites that often cause behavioral, pathogenic, and evolutionary effects on their hosts. Interactions between ectoparasites and avian hosts, especially migrating taxa, may influence avian pathogen spread in tropical and temperate ecosystems and affect long-term survival, fitness and reproductive success. The purpose of this study was to characterize the vector-associated microbiome of ectoparasitic louse flies feeding on migrating raptors over the fall migration period. Surveys for louse flies occurred during fall migration (2015–2016) at a banding station in Pennsylvania, United States; flies were collected from seven species of migrating raptors, and we sequenced their microbial (bacteria and archaea) composition using high-throughput targeted amplicon sequencing of the 16S rRNA gene (V4 region). All louse flies collected belonged to the same species, *Icosta americana*. Our analysis revealed no difference in bacterial communities of louse flies retrieved from different avian host species. The louse fly microbiome was dominated by a primary endosymbiont, suggesting that louse flies maintain a core microbial structure despite receiving blood meals from different host species. Thus, our findings highlight the importance of characterizing both beneficial and potentially pathogenic endosymbionts when interpreting how vector-associated microbiomes may impact insect vectors and their avian hosts.

## Introduction

Each autumn millions of raptors migrate from their breeding grounds in North America to overwintering areas in South America, following well-established routes created by geographical features and ideal weather conditions [1–3]. One of the major migratory routes in North America, the Atlantic Flyway, ranges from northeastern Canada to the southern United States. The Atlantic Flyway extends along the Atlantic Coast and the Appalachian Mountains and is where over 16 species of raptors converge with songbirds and waterfowl, as they make their way south to their overwintering habitats. This migratory period is one of the riskiest stages in the annual cycle due to the direct physiological challenges (i.e., energetically demanding, depletion of fat reserves) [4], higher mortality rates [5], and potential pathogenic infections acquired from insects that may influence the health and overall survivorship of these birds [6, 7]. Flying longer distances, between the northern and southern hemispheres, raptors may increase the potential for intra- and intercontinental spread of ectoparasitic insects and their symbionts, both beneficial and pathogenic [8–10]. Understanding which traits influence the vectoral capacity of raptor ectoparasites may elucidate new threats to declining raptor populations and how such threats may be mitigated.

Dipteran species that possess intertwined life histories with avian populations can have a multitude of pathogenic, ecological, and evolutionary effects on the avian host [11, 12] by serving as infectious hosts for avian and human pathogens [e.g., West Nile Virus (WNV)] or negatively impacting the avian host (e.g., anemia, hyperkeratosis, and myiasis) [7, 13, 14]. From an ecological perspective, ectoparasite and avian host interactions can influence pathogen spread from temperate to tropical ecosystems, and vice versa, especially within taxa migrating across different geographic regions [15, 16]. For example, the highly pathogenic influenza strain, H5N1, moved from Asia to the Middle East, Europe, and Africa via globally distributed wild birds that are reservoirs for the virus [17, 18]. In addition to hosting pathogens, ectoparasites can also have indirect evolutionary effects on fitness and reproductive success (e.g., influence mate selection) in infected birds, ultimately causing feather damage, reduced egg production, and increased mortality [19–21].

Louse flies (Diptera: Hippoboscidae) are a group of Brachyceran flies with 223 species that parasitize birds and mammals, and are common ectoparasites on many raptors around the world [22]. Both hippoboscid sexes require a blood meal and can survive beneath the feathers of their host for several months [23]. Infestations of louse flies can range from one individual to more than ten [24, R. McCabe pers. observation] on a single bird at one time. Some louse fly species are highly mobile, flying from host-to-host, with the ability to abandon a newly dead host for one that is alive [24, 25]. One species of Hippoboscidae, *Icosta americana*, with a Nearctic and Neotropical distribution [22], were collected off wild raptors submitted to a rehabilitation center in New Jersey, United States, in 2003 and tested positive for WNV viral RNA, implicating the transmission of WNV between host and vector [24].

The vectoral capacity of louse flies paired with the costs associated with seasonal migration may pose health risks to raptors and ultimately impact survival [26, 27]. This combination may be especially detrimental to juvenile raptors that have high first-year mortality rates (i.e., > 50%) [28]. A first step to understanding how ectoparasitic louse flies may impact raptor populations is to characterize the vector-associated microbiome of blood feeding louse flies collected from wild-caught migratory raptors. This first step may ultimately lead to identification of pathogen transmission and persistence from insects to birds through vector-associated microbiome characteristics. Our objectives included: 1) the quantification of louse fly abundance on different species of migrating raptors over time, and 2) the characterization of bacterial and archaeal communities found within blood feeding louse flies, including beneficial and potentially pathogenic microbial taxa.

## Materials and methods

### Sample collection and preparation

Louse flies were collected from migrating raptors over a two-year period between September–November 2015 and 2016 at the Little Gap Banding Station in Northampton County, Pennsylvania, United States (40° 48' N, 75° 32' W), as part of an annual effort to monitor migrating raptor populations. Birds were trapped by authorized personnel using either mist nets or bow traps and a harnessed Rock Pigeon (*Columba livia*) [29] and immediately removed from traps and secured for processing, according to the U.S. Geological Survey Bird Banding Laboratory. A 90-second visual and physical survey (parting feathers) of the dorsal and ventral surface of the bird was conducted in order to identify the presence or absence of louse flies. A federal permit for the trapping, handling, and processing of birds was provided by the U.S. Geological Survey Bird Banding Laboratory (Permit #21371–G. Lahr, Little Gap Banding Station), and carried out in strict accordance with The Bander’s Code of Ethics, approved by the North American Banding Council. Being a non-invasive procedure, no special permission was needed for the collection of louse flies off of raptor hosts. All louse flies from an individual were collected by hand or with forceps, immediately submerged in 100% ethanol (one tube per individual bird), stored at room temperature, and later transported to the laboratory for DNA extraction. Louse flies were identified to species level prior to extraction [22]. The age, sex, weight, species of raptor, date, and time of capture were documented for all birds from which louse flies were collected (S1 Table). Not all individuals being banded were examined for flies and not all flies were captured from each individual due to time constraints.

### DNA extraction and sequencing

To identify the impact of host species and temporal variation on internal bacterial communities, collected louse flies were pooled by bird species and collection date, resulting in 1–5 individuals per sample (*n* = 2 louse flies/bird). A sub-sample of flies were sequenced from each species throughout the fall migration of 2015–2016 (Table 1). For purposes of decontamination, prior to DNA extraction louse flies received a two-minute wash in a 10% bleach solution. To facilitate homogenization of the louse flies prior to extraction, louse flies were dissected (i.e. body cut into pieces) with flame-sterilized scissors. DNA extractions were performed following the DNA PowerSoil DNA Isolation Kit (Qiagen) manufacturer’s recommended protocol. Individual DNA yields were quantified using the Qubit ^®^ dsDNA High Sensitivity assay and Qubit 2.0, and resulting DNA was stored at -20 °C prior to library preparation.

**Table 1:** Sequenced samples of louse flies collected from migrating raptors between September–November in 2015 and 2016 in Pennsylvania, United States. Comparison of louse flies collected from raptor species during each month of the 2015–2016 survey period. In parentheses is the total number of louse flies sequenced for microbiome analysis for each month and year per raptor host species.

	September		October		November		Total Samples	
Species	2015	2016	2015	2016	2015	2016	2015	2016
Bald Eagle (BAEA); *n* = 1 bird	1(1)	-	-	-	-	-	1	0
Broad-winged Hawk (BWHA); *n* = 4 birds	1(1)	4(4)	-	-	-	-	1	4
Cooper's Hawk (COHA); *n* = 10 birds	3(5)	-	2(3)	1(1)	-	1(1)	5	2
Northern Goshawk (NOGO); *n* = 2 birds	-	-	-	-	-	2(3)	0	2
Red-shouldered Hawk (RSHA); *n* = 1 bird	-	-	-	-	-	1(2)	0	1
Red-tailed Hawk (RTHA); *n* = 20 birds	2(2)	7(15)	-	2(5)	1(1)	4(8)	3	13
Sharp-shinned Hawk (SSHA); *n* = 17 birds	4(5)	2(3)	-	2(4)	-	-	4	4

Library construction and sequencing (Illumina MiSeq, 2 x 250 bp paired-end reads) was performed as described by [30] at the Michigan State University Genomics Core Facility. Dual indexed primers 515f/806r (5′-GTGCCAGCMGCCGCGG-3′, 5′-TACNVGGGTATCTAATCC-3′) were used to amplify the V4 region of the 16S rRNA gene [30, 31]. Prior to sequencing, the PCR products were normalized using SequalPrep^®^ normalization plates (Invitrogen) and cleaned with AMPureXP magnetic beads (Beckman Coulter Life Sciences). Custom sequencing and index primers were added as previously described [31].

#### Bioinformatic processing

After demultiplexing (Bcl2fastq v 2.19.1, Illumina), the reads were quality filtered using DADA2 in QIIME2 (v 2019.7) before chimeric sequences were removed [32, 33]. To taxonomically assign sequencing reads a Naïve Bayes classifier was trained using the V4 16S rRNA region and the SILVA database (99% confidence, v 132) [34]. Filtered sequencing reads were then assigned to taxonomic groups using the trained classifier and default settings in QIIME2. Mitochondrial and chloroplast reads were removed prior to rooted phylogenetic tree construction using FastTree (v 2.0) [35] and MAFFT (v 7.0) [36]. Alpha-diversity [Shannon diversity, and Faith’s phylogenetic diversity (Faith’s PD)] and beta-diversity (Jaccard and weighted UniFrac distance) metrics were also calculated in QIIME2 using default settings [37]. Sequencing files for this study have been deposited in the NCBI database under the accession number PRJNA574458.

### Statistical analyses

We tested for effects of year on diversity (Faith’s PD, Shannon, *P* > 0.05) and taxonomic composition (Kruskal-Wallis, *P* > 0.05), and found no statistically significant difference, thus 2015 and 2016 were pooled for further analyses. To determine if sampling date or raptor host species impacted the diversity of the internal microbiome present in louse flies, differences in alpha-diversity metrics were tested using Kruskal-Wallis tests in R (v 3.5.2) with a False Discovery Rate (FDR) correction for multiple samples [38]. Pairwise comparisons among species were conducted using a Mann-Whitney test with an FDR correction in R. Similarly, differences in the relative abundance of bacterial phyla and genera among host species were tested with Kruskal-Wallis tests. To limit the potential for spurious results from taxa with very low abundance, only taxa which comprised greater than 1% of the total bacterial community were compared at the genus level. To test if temporal effects impacted the ectoparasite bacterial communities [i.e., early (September) or late (November) migrating raptors], beta-diversity metrics were analyzed using PERmutational Multivariate Analysis Of Variance (PERMANOVA) after checking for homogeneity of variances between samples groups in the vegan package (v 2.5–2) [39]. Figures were created using a combination of the vegan, phyloseq (v 1.24.1), and ggplot2 (v 3.0.0) libraries in R [40, 41]. All code used in this analysis is available at: https://github.com/BenbowLab/MUHippoboscid.

## Results

### Raptor host and ectoparasite species

A total of 125 louse flies (*n* = 39 flies in 2015, 86 flies in 2016) were collected from seven species of raptors (S1 Table). All flies collected were identified as the same species, *Icosta americana* (Diptera: Hippoboscidae) (Leach 1817), a Nearctic and Neotropical species chiefly found on Accipitridae, Phasianidae, and Strigidae [22]. The host species of migrating raptors captured and louse flies collected from included: Bald Eagle (*Haliaeetus leucocephalus*), Broad-winged Hawk (*Buteo platypterus*), Cooper’s Hawk (*Accipiter cooperii*), Northern Goshawk (*Accipiter gentilis*), Red-shouldered Hawk (*Buteo lineatus*), Red-tailed Hawk (*Buteo jamaicensis*), and Sharp-shinned Hawk (*Accipiter striatus*).

### Bacterial communities

**Fig 1:**
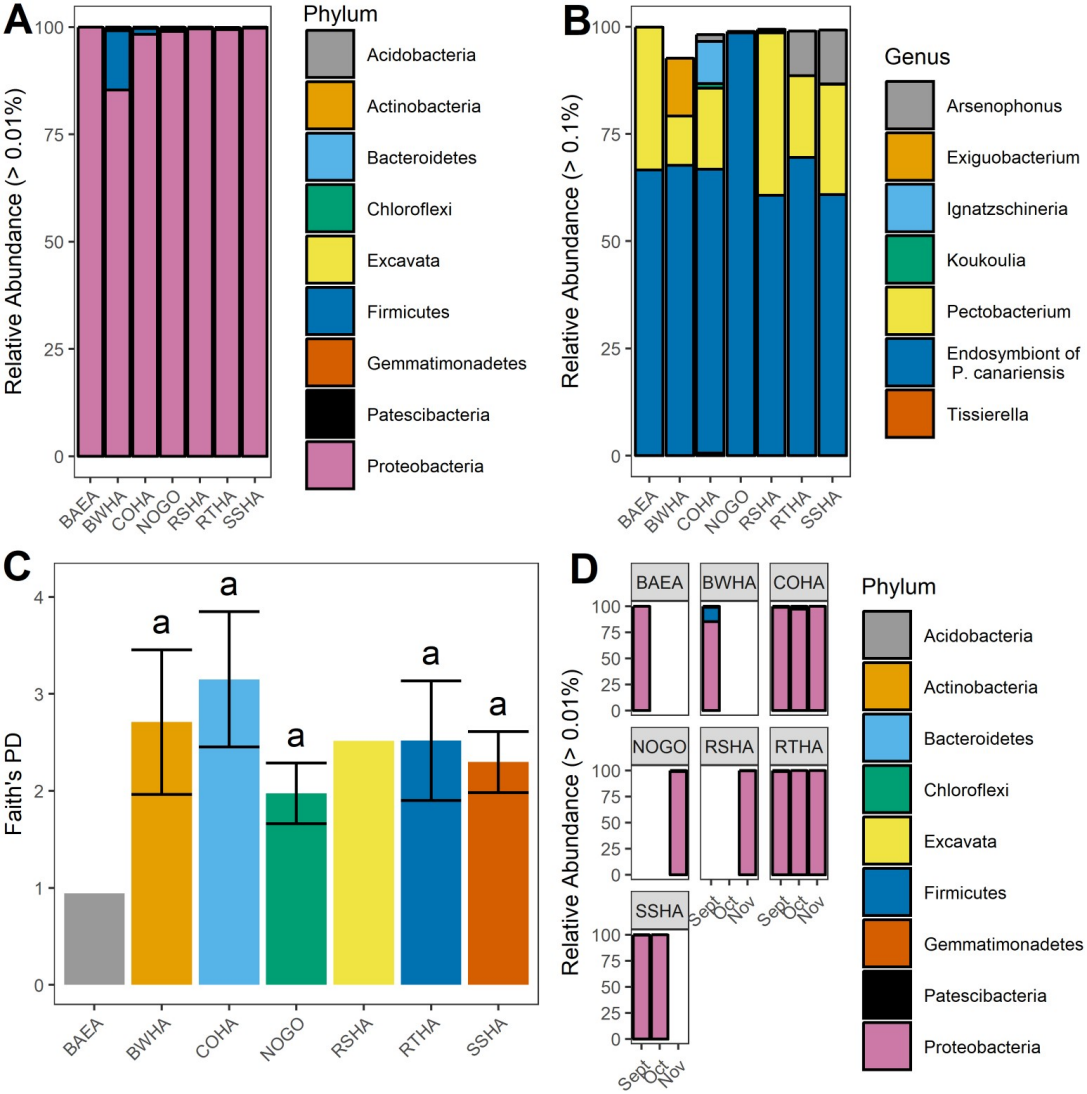
Bacterial communities of louse flies collected from migrating raptors during fall migration 2015–2016. (A) Phylum level relative abundance of bacterial communities of louse flies collected from seven raptor host species. Only phyla greater than 0.01% of the total relative abundance are shown. (B) Genus level relative abundance of bacterial communities of louse flies collected from raptor host species. Only genera greater than 0.1% of the total relative abundance are shown. (C) Mean Faith’s Phylogenetic Diversity (PD) of louse fly internal bacterial communities by raptor host species. Error bars represent SEM. Identical letters indicate nonsignificant pairwise comparisons between species with greater than three samples (Mann-Whitney test, FDR correction). (D) Phylum level bacterial relative abundance of louse flies by sampling month (2015 and 2016 combined) for each raptor host species. Abbreviations for raptor host species are as follows: Bald Eagle (BAEA), Broad-winged Hawk (BWHA), Cooper’s Hawk (COHA), Northern Goshawk (NOGO), Red-shouldered Hawk (RSHA), Red-tailed Hawk (RTHA), and Sharp-shinned Hawk (SSHA).

A total of 40 samples comprising 64 louse flies were sequenced (Table 1) resulting in 2,490,039 reads and 339 sequence variants after filtering. To limit bias due to differing read library sizes [42], samples were rarified to 5,000 reads based on alpha rarefaction plots. We tested for effects of year on alpha-diversity (Faith’s PD, Shannon Diversity, *P* > 0.05) and taxonomic composition (Kruskal-Wallis, *P* > 0.05), and found no statistical difference, thus 2015 and 2016 were pooled for further analyses. The phylum Proteobacteria dominated the internal microbiome of all louse flies but one sample, and comprised greater than 97% of the total bacterial community regardless of which raptor host species they were collected from (Fig 1A). Firmicutes comprised 2% of the community, while the remaining 1% of phyla included a combination of Bacteroidetes, Actinobacteria, and Acidobacteria.

**Fig 2:**
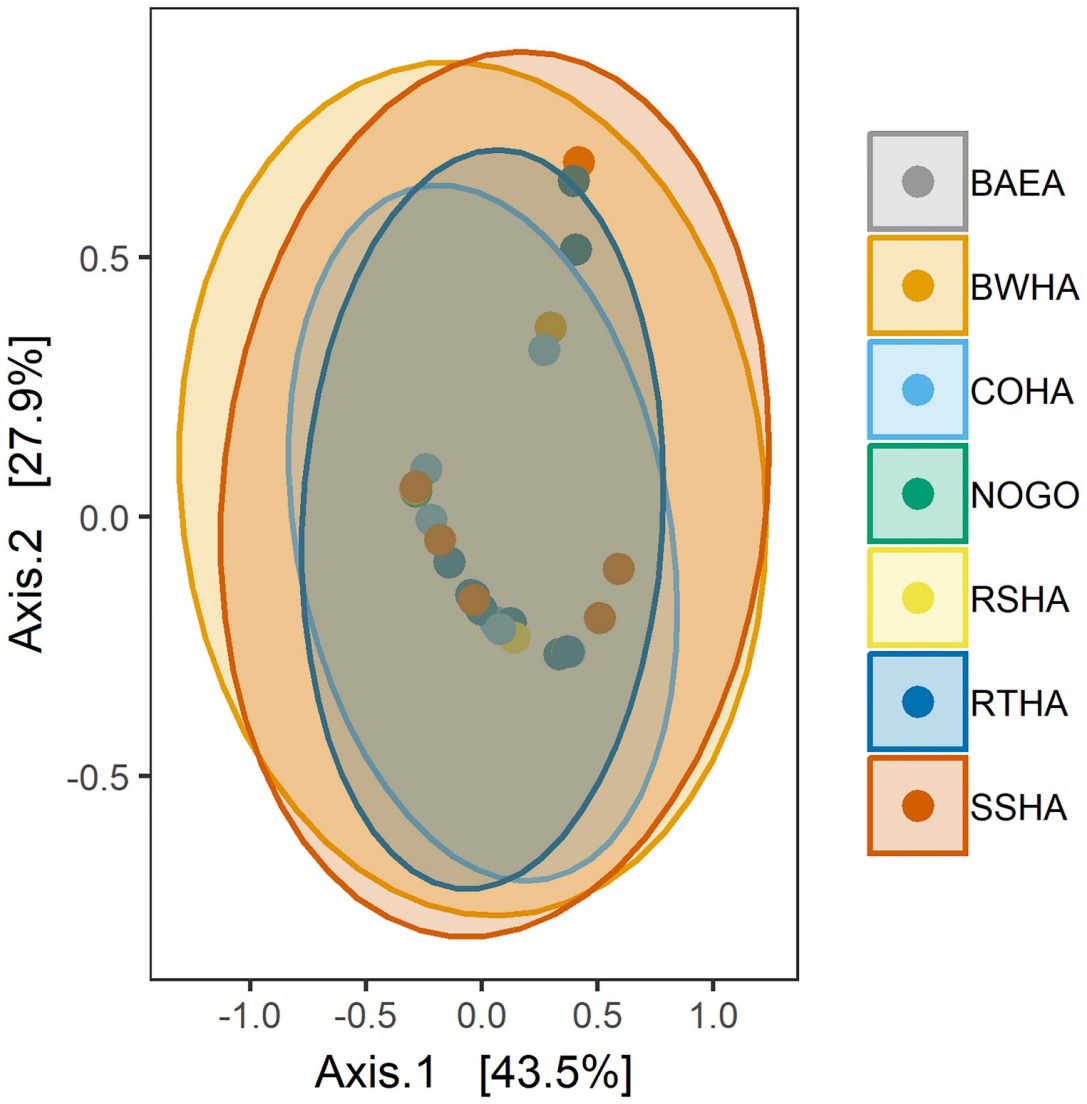
Principal Coordinates Analysis (PCoA) of beta-diversity (Jaccard) of louse fly bacterial communities collected from raptor host species. Ellipses represent 95% confidence interval for the mean of each raptor species host group. Abbreviations for raptor host species are as follows: Bald Eagle (BAEA), Broad-winged Hawk (BWHA), Cooper’s Hawk (COHA), Northern Goshawk (NOGO), Red-shouldered Hawk (RSHA), Red-tailed Hawk (RTHA), and Sharp-shinned Hawk (SSHA).

The family Enterobacteriaceae comprised greater than 94% of the bacterial communities of *I*. *americana*, with a specific taxon classified as “primary endosymbiont of the pigeon louse fly (*Pseudolynchia canariensis*)” representing 68.13% (+/- 5.34 SEM) of the total community (Fig 1B). In addition to the endosymbiont of *Ps*. *canariensis*, bacterial genera detected in lower relative abundances included: *Pectobacterium* (19.31% +/- 3.99 SEM), *Arsenophonus* (6.96% +/- 3.74 SEM), *Ignatzschineria* (1.74% +/- 1.72 SEM), and *Exiguobacterium* (1.69% +/- 1.69 SEM). Among louse flies from different host species there were no statistical differences in the relative abundance of any bacterial taxa at the phylum, family or genus level (Kruskal-Wallis, *P* > 0.70). When testing for differences in beta-diversity, weighted UniFrac distance had significantly different homogeneity of variances between host species (PERMANOVA, *P* < 0.01) while Jaccard distance did not (*P* > 0.05), so Jaccard distance was used for subsequent analysis as it did not violate the assumptions of PERMANOVA. Similarly, there were no differences in alpha-diversity (Faith’s PD, Shannon, Kruskal-Wallis, *P* > 0.5, Fig 1C) or beta-diversity (Jaccard, PERMANOVA, *P* > 0.90) metrics among louse flies on different avian host species. There were no statistically significant temporal differences [early (September) vs. late (November)] in taxonomic composition (Kruskal-Wallis, *P* > 0.05, Fig 1D), alpha-diversity metrics (Kruskal-Wallis, *P* > 0.05) or beta-diversity (PERMANOVA, *P* = 0.54, Fig 2).

## Discussion

Few studies have examined the microbiome of raptor hosts, much less the microbiomes of their ectoparasites [43–45]. To our knowledge, this study is the first to characterize the bacterial communities of louse flies collected off of various species of migrating raptors in North America. Surprisingly, only one species of louse fly, *I*. *americana*, was found across seven raptor host species during fall migration. The relative bacterial abundance of *I*. *americana* was dominated by the phylum Proteobacteria (97%) and to a lesser extent Firmicutes (2%), regardless of which raptor host species they were feeding on. Proteobacteria and Firmicutes have also been identified as abundant bacteria in other microbiome studies of avian tick ectoparasites [46], carrion beetles [47], as well as other birds [44, 48]. Similarly, 80% of the bacterial sequences detected from cloacal swabs of Bald Eagles captured from the coastal beaches of Washington and Oregon, were Firmicutes and Proteobacteria [49], suggesting these prominent phyla can be found across a range of host species (e.g., from insects to birds).

From the family Enterobacteriaceae, the most predominant genus found in the louse fly microbiome collected from all seven raptor host species was classified as a “primary endosymbiont of the pigeon louse fly (*Ps*. *canariensis*)”. Another study characterized an endosymbiotic bacterium related to the *Arsenophonus* genus, Candidatus *Arsenophonus arthropodicus*, from the tissues of the pigeon louse fly [50]. In our study, we found a higher abundance (i.e., > 50% across species) of the primary endosymbiont from *Ps*. *canariensis*, thus suggesting a similar finding [50] with the presence of *Arsenophonus sp*. observed in our samples. Other arthropod studies have identified *Arsenophonus*, which forms a distinct monophyletic clade of arthropod endosymbionts in the gamma subdivision of Proteobacteria [50], in ticks [51], whiteflies [52], aphids [53, 54], psyllids [55], and parasitoid wasps [56]. Primary endosymbionts have a long evolutionary history with their hosts insects and may play an unknown beneficial role in the host [50].

Louse flies and other closely related hematophagous insects maintain an obligate symbiosis with bacterial species (e.g., Candidatus *Arsenophonus arthropodicus* [50], Candidatus *Arsenophonus lipopteni* [57]). In many species, these interactions are highly specialized with a single to a few symbionts [57]. It has been hypothesized that this relationship is a result of the need for insects to obtain vitamins (e.g., vitamin B) that they do not acquire from their blood meals, but instead are produced by the symbiotic bacteria [58, 57], and thus compounds in the host blood may have an effect on the community composition of the louse fly microbiome. Enterobacteriaceae are pervasive and considered part of the autochthonous intestinal flora of some species of birds, but not normally found in raptor species including hawks and vultures (Accipitriformes), falcons (Falconiformes), or owls (Strigiformes) [59]. Though we did not sample the microbiota of raptor host species in this study, the consistent dominance of this primary endosymbiont in all the louse flies regardless of which raptor host species they were collected from suggests this bacterium is specific to louse fly hosts and not acquired through blood meals.

*Pectobacterium* (Phylum: Proteobacteria, Family: Enterobacteriaceae) was the second most abundant bacterial genus found in louse flies in this study, but with < 1% found in louse flies collected from Northern Goshawks. These bacteria are related to soft-rot disease in plants [60] where they infect a range of hosts by consuming cellular nutrients and degrading plant tissues [61]. *Pectobacterium* contributes to its insect host’s nutrition by fixing atmospheric nitrogen, an essential element for physiological activities such as reproduction and development [62]. *Pectobacterium* sp. have been associated with bark beetles (*Dendroctonus* spp.) in the United States and Mexico [63], Mediterranean fruit flies (Tephritidae: *Ceratitis capitate*) [64], Brazilian fruit flies (Drosophilidae: *Drosophila* spp.) [60], and wood-boring beetles in Italy [65]. Associations with nitrogen fixing bacteria, such as *Pectobacterium*, among tephritid and drosophilid Diptera are critical for fly development and reproduction [64]. Because louse fly larvae feed on nourishing “milk” glands from within female louse flies [23], it is possible that this endosymbiont may be beneficial to louse fly larvae in the processing of nitrogen-rich blood as a result of adults feeding on migrating raptors. If this is true, this is the first time a bacterial endosymbiont has been associated with a hematophagic (fluid-feeding) vector of potentially harmful endoparasites to birds.

The bacterial genera *Exiguobacteria* (Firmicutes) and *Ignatzschineria* (Firmicutes) comprised the remaining genera (< 4%) found within *I*. *americana* in this study. The genus *Exiguobacterium* is a diverse group of pigmented gram-positive bacteria [66] that possess stress-responsive genes allowing them to occupy and thrive in diverse ecological niches and can cause bacteremia in immunocompromised hosts [59, 67]. This bacterium has been found in the microbiomes of herbivorous insects such as leafhoppers (*Macrosteles sexnotatus*) [68] and grasshoppers (*Sathrophyllia femorata*) [69], as well as in the midguts of the filter-feeding larval mosquito species *Aedes japonicus*, *A*. *triseriatus*, *and Culex pipiens* [70]. Interestingly, we document the presence of *Exiguobacterium* in hematophagic louse flies collected from Broad-winged Hawks (13.5%) only and not in the other six avian host species. Broad-winged Hawks are forest nesting raptors [71] and perhaps this association with louse flies may be connected to the breeding habitat differences between Broad-winged Hawks and the other raptor host species in this study. The other Firmicute bacterium found within louse flies in this study was *Ignatzschineria sp*. This gram-negative bacterium is strongly associated with carrion-breeding flies, especially the obligate parasitic fly, *Wohlfahrtia magnifica* (Diptera: Sarcophagidae) responsible for myiasis [72, 73], black soldier flies (*Hermetia illucens*) (Diptera: Stratiomyidae) [74], and blow flies (Diptera: Calliphoridae) [75]. We found a higher percent of *Ignatzschineria* in louse flies of Cooper’s Hawks (9.83%). Thus, further research is necessary to determine if the presence of these Firmicutes in ectoparasitic louse flies is related to the louse fly larval habitat, and if this factor influences the ability for the bacterium to be acquired by louse flies from host blood meals.

Louse fly reproduction is tied to the host nest where the larva pupates almost immediately after being deposited into the nest [23], thus infestations are common among hatch year (juvenile) birds as they provide a food source for newly emerged louse flies of that year [23, 24]. The infestation occurs in the nest, but the louse flies will remain and feed on the young birds throughout the year [24]. This relationship may explain why louse flies in our study were predominately collected off of hatch year host raptor species (S1 Table) during fall migration, although this warrants further investigation. Host switching was detected in *Olfersia spinifera*, a species of louse fly that parasitizes the Great Frigatebird (*Fregata minor*), when researchers discovered that the blood meal from the louse fly did not match the blood from the host [76]. The unknown life span of louse flies (i.e., probably surviving for several months) [23] in combination with dispersal of hatch year birds from their natal sites and potential for host-switching warrants further investigation of vector traits, such as its microbiome, that help facilitate the movement of louse flies among hosts and across geographical boundaries.

Vector-associated bacterial taxa can be beneficial to their insect hosts in multiple ways, including dietary specialization to an exclusive blood diet [77], development [78], and immunity [79]. Recent studies have also highlighted the importance of the microbiome in the context of host pathogen transmission processes [80–82]. The genetic ability for an insect to transmit pathogens is measured in terms of its vector competence and this competence depends on the proficiency of the host insect’s immune responses, which may be facilitated through host immune priming by the host-associated microbiota [80]. For example, natural gut microbiota stimulated the same innate immune pathway that regulates viral resistance in mosquitoes [83]. The gut represents the primary pathogen entry point during each blood meal, therefore gut microbiota may directly mediate pathogen colonization and extrinsic incubation time (i.e., time necessary for pathogens to development inside the insect vector) [84], as observed in ticks fed on antibiotic-treated mice that exhibited modified gut microbiota composition and lower *Borrelia burgdorferi* colonization rates [85].

Previous studies have hypothesized the current consensus of global climate change may be promoting increased numbers of avian blood parasites and pathogens [86, 87], which depend on the abundance and distribution of vectors, leading to an increase in documented ectoparasitic loads on avian hosts [88, 89]. The prevalence of louse flies collected from nearly half (i.e., seven out of the 16) of the migrating raptor species in Pennsylvania demonstrates the potential for widespread distribution of louse flies. The seven host species for the studied louse flies migrate from Canada down to Mexico (e.g., Bald Eagle, Northern Goshawk, Red-shouldered Hawk), Central America (e.g., Red-tailed Hawk, Sharp-shinned Hawk, Cooper’s Hawk), and as far south as the northern tip of Argentina, South America (e.g., Broad-winged Hawk). The geographical distribution of *I*. *americana* ranges from 49° N to 30° S [90], suggesting the intercontinental dispersal of louse flies via their migrating hosts. The first record of *I*. *americana* on a breeding American Kestrel (*Falco sparverius*) in central Argentina (i.e., 36° S) indicates southern range expansion for this dipteran species [7].

In conclusion, *I*. *americana* was the only species of ectoparasitic louse fly found feeding on the seven species of raptor hosts sampled. We found no difference in the bacterial communities of louse flies regardless of which raptor species they were collected from. The louse fly microbiome was dominated by a primary endosymbiont, suggesting that louse flies may maintain a core microbial structure despite receiving blood meals from different host species. Based on this finding, we suspect this primary endosymbiont plays an unknown beneficial function for its insect host, such as increased vector competence. We suggest that future work should include isolating the primary endosymbiont from *I*. *americana* and test for strains of specific pathogens found within ectoparasite hosts. Future studies should sample for louse flies on raptors in South America, both residents and migrants, to determine if there is an overlap in vector-associated bacterial communities between continents.

## Supporting information

S1 Table Raptor host species that louse flies were collected off of in 2015–2016 from a migration banding station in Pennsylvania, United States. Age categories of raptor species included: 1) HY = Hatch Year, 2) SY = Second Year, 3) ASY = After Second Year, and 4) PB2 or PB3 = Prebasic Molt. Sex categories of raptor species included: 1) F = Females, 2) M = Males, and 3) U = Unknown. Abbreviations for raptor species are as follows: Bald Eagle (BAEA), Broad-winged Hawk (BWHA), Cooper’s Hawk (COHA), Northern Goshawk (NOGO), Red-shouldered Hawk (RSHA), Red-tailed Hawk (RTHA), and Sharp-shinned Hawk (SSHA).
(DOCX)

## References

[1] Titus T, Mosher JA. The influence of seasonality and selected weather variables on autumn migration of three species of hawks through the Central Appalachians. Wilson Bull. 1982; 94: 176–184.

[2] Bildstein KL. Migrating raptors of the world: their ecology and conservation. Ithaca: Cornell University Press; 2006.

[3] Goodrich LJ, Smith JP. Raptor migration in North America State of North America’s Birds of Prey. Cambridge and Washington, DC: Nuttall Ornithological Club and American Ornithologists’ Union; 2008.

[4] McWilliams SR, Guglielmo C, Pierce B, Klaassen M. Flying, fasting, and feeding in birds during migration: a nutritional and physiological ecology perspective. J Avian Biol. 2004; 35(5): 377–393.

[5] Klaassen RH, Hake M, Strandberg R, Koks BJ, Trierweiler C, Exo KM, et al When and where does mortality occur in migratory birds? Direct evidence from long-term satellite tracking of raptors. J Anim Ecol. 2014; 83(1): 176–84. 10.1111/1365-2656.12135 24102110

[6] Gancz AY, Barker IK, Lindsay R, Dibernardo A, McKeever K, Hunter B. West Nile Virus Outbreak in North American Owls, Ontario, 2002. Emerg Infect Dis. 2004; 10(12): 2136–2142. 10.3201/eid1012.040167 PMC3323370 15663850

[7] Liébana MS, Santillan MA, Cicchino AC, Sarasola JH, Martinez P, Cabezas S, et al Ectoparasites in free-ranging American kestrels in Argentina: implications for the transmission of viral diseases. J Raptor Res. 2011; 45: 335–341.

[8] Varma MGR. Mites (family Trombiculidae) parasitizing birds migrating from Africa to Europe. Bull World Health Organ. 1964; 31(3): 411. 14267750 PMC2555110

[9] Bjöersdorff A, Bergström S, Massung RF, Haemig PD, Olsen B. Ehrlichia-infected ticks on migrating birds. Emerg Infect Dis. 2001; 7(5): 877 10.3201/eid0705.017517 11747702 PMC2631880

[10] Ioannou I, Chochlakis D, Kasinis N, Anayiotos P, Lyssandrou A, Papadopoulos B, et al Carriage of Rickettsia spp., *Coxiella burnetii* and *Anaplasma* spp. by endemic and migratory wild birds and their ectoparasites in Cyprus. Clinical Microbiology and Infection. 2009; 15: 158–160. 10.1111/j.1469-0691.2008.02207.x 19281460

[11] Brown CR, Brown MB, Rannala B. Ectoparasites Reduced Long-Term Survival of their Avian Host. Proc R Soc Lond B.1995; 262: 313–319.

[12] Barber I, Dingemanse NJ. Parasitism and the evolutionary ecology of animal personality. Phil Trans R Soc B. 2010; 365: 4077–4088. 10.1098/rstb.2010.0182 21078659 PMC2992744

[13] Turrell M. Arthropod-related viruses of medical and veterinary importance In: Mullen GR, Durden LA, editors. Journal of Medical and Veterinary Entomology, second edition. San Diego: Academic Press; 2009 pp. 55–564.

[14] Hunter DB, Rohner C, Currie DC. Mortality in fledgling great horned owls from black fly hematophaga and leucocytozoonosis. J Wild Dis. 1997; 33(3): 486–91. 10.7589/0090-3558-33.3.486 9249694

[15] Rvachev LA, Longini IM. A mathematical model for the global spread of influenza. Math Biosci. 1985; 75: 3–23.

[16] Fuller T, Bensch S, Muller I, Novembre J, Perez-Tris J, Ricklefs RE, et al The Ecology of Emerging Infectious Diseases in Migratory Birds: An Assessment of the Role of Climate Change and Priorities for Future Research. Eco Health. 2012; 9: 80–88. 10.1007/s10393-012-0750-1 22366978

[17] Peterson AT, Benz BW, Papeş M. Highly pathogenic H5N1 avian influenza: entry pathways into North America via bird migration. PLoS one. 2007; 2(2): e261 10.1371/journal.pone.0000261 17330144 PMC1803015

[18] Deliberto TJ, Swafford SR, Nolte DL, Pedersen K, Lutman MW, Schmit BB, et al Surveillance for highly pathogenic avian influenza in wild birds in the USA. Integr Zool. 2009; 4(4): 426–39. 10.1111/j.1749-4877.2009.00180.x 21392315

[19] Philips JR. What’s bugging your birds? Avian parasitic arthropods. Wildl Rehab. 1990; 8: 155–203.

[20] Philips JR. Pathology- Ectoparasites In: Bird DM, Bildstein KL, editors. Raptor Research and Management Techniques. Surrey: Hancock House Publishers; 2007 pp. 311–312.

[21] Jahantigh M, Esmailzade Dizaji R, Teymoori Y. Prevalence of external parasites of pigeon in Zabol, southeast of Iran. Journal of parasitic diseases: official organ of the Indian Society for Parasitology. 2016; 40(4): 1548–1551. 27876980 10.1007/s12639-015-0725-6 PMC5118351

[22] Maa TC. A revised checklist and concise host index of Hippoboscidae (Diptera). Pacific Insects Monograph. 1969; 20: 261–299.

[23] Baker JR. A review of the role played by the Hippoboscidae (Diptera) as vectors of endoparasites. J Parasitol. 1967; 53(2): 412–418. 5336850

[24] Farajollahi A, Crans WJ, Nickerson D, Bryant P, Wolf B, Glaser A, et al Detection of West Nile virus RNA from the louse fly *Icosta americana* (Diptera: Hippoboscidae). J Am Mosquito Contr. 2005; 21(4): 474–477. 10.2987/8756-971X(2006)21[474:DOWNVR]2.0.CO;2 16506578

[25] Bequaert JC. The Hippoboscidae or louse-flies (Diptera) of mammals and birds. Part I. Structure, physiology and natural history. Entomol AM-NY. 1953; 33: 211–442.

[26] Remple JD. Intracellular hematozoa of raptors: a review and update. J Avian Med Surg. 2004; 18(2): 75–89.

[27] Levin II, Valkiūnas G, Santiago-Alarcon D, Cruz LL, lezhova TA, O’Brien SL, et al Hippoboscid-transmitted Haemoproteus parasites (Haemosporida) infect Galapagos Pelecaniform birds: Evidence from moleculas and morphological studies, with a description of Haemoproteus iwa. Int J Parisitol. 2011; 41(10): 1019–1027. 10.1016/j.ijpara.2011.03.014 21683082

[28] Newton I. Population ecology of raptors. Vermillion: Buteo Books; 1979.

[29] Berger DD, Hamerstrom F. Protecting a trapping station from raptor predation. J Wildl Manag. 1962; 26(2): 203–206.

[30] Caporaso JG, Lauber CL, Walters WA, Berg-Lyons D, Lozupone CA, Turnbaugh PJ, et al Global patterns of 16S rRNA diversity at a depth of millions of sequences per sample. PNAS. 2011; 108: 4516–4522. 10.1073/pnas.1000080107 20534432 PMC3063599

[31] Kozich JJ, Westcott SL, Baxter NT, Highlander SK, Schloss PD. Development of a dual-index sequencing strategy and curation pipeline for analyzing amplicon sequence data on the MiSeq Illumina sequencing platform. Appl Environ Microbiol. 2013; 79: 5112–5120. 10.1128/AEM.01043-13 23793624 PMC3753973

[32] Callahan BJ, McMurdie PJ, Rosen MJ, Han AW, Johnson AJA, Holmes SP. DADA2: high-resolution sample inference from Illumina amplicon data. Nat Methods. 2016; 13: 581 10.1038/nmeth.3869 27214047 PMC4927377

[33] Bolyen E, Rideout JR, Dillon MR, Bokulich NA, Abnet C, Al-Ghalith GA, et al Reproducible, interactive, scalable and extensible microbiome data science using QIIME 2. Nature biotechnology 2019; 37(8): 852–857. 10.1038/s41587-019-0209-9 31341288 PMC7015180

[34] Bokulich NA, Kaehler BD, Rideout JR, Dillon M, Bolyen E, Knight R, et al Optimizing taxonomic classification of marker-gene amplicon sequences with QIIME 2’s q2-feature-classifier plugin. Microbiome. 2018; 6: 90 10.1186/s40168-018-0470-z 29773078 PMC5956843

[35] Price MN, Dehal PS, Arkin AP. FastTree 2–approximately maximum-likelihood trees for large alignments. PloS one. 2010; 5: e9490 10.1371/journal.pone.0009490 20224823 PMC2835736

[36] Katoh K, Standley DM. MAFFT multiple sequence alignment software version 7: improvements in performance and usability. Mol Biol Evol. 2013; 30: 772–780. 10.1093/molbev/mst010 23329690 PMC3603318

[37] Faith DP, Baker AM. Phylogenetic diversity (PD) and biodiversity conservation: some bioinformatics challenges. Evolutionary bioinformatics. 2006; 2: 117693430600200007. PMC2674678 19455206

[38] R Core Team. R: A language and environment for statistical computing; 2018 [cited 5 Aug 2019]. R Foundation for Statistical Computing [online]. https://www.R-project.org/.

[39] Oksanen J, Blanchet FG, Kindt R, Legendre P, Minchin PR, O’hara R, et al. Package ‘vegan’. Community ecology package, version 2; 2015 [cited 18 October 2019].

[40] McMurdie PJ, Holmes S. Phyloseq: An R Package for Reproducible Interactive Analysis and Graphics of Microbiome Census Data. PLoS one. 2013; 8: e61217 10.1371/journal.pone.0061217 23630581 PMC3632530

[41] Wickham H. ggplot2: elegant graphics for data analysis; 2016 [cited 30 Aug 2019].

[42] Weiss S, Xu ZZ, Peddada S, Amir A, Bittinger K, Gonzalez A, et al 2017. Normalization and microbial differential abundance strategies depend upon data characteristics. Microbiome. 2017; 5(1): 27 10.1186/s40168-017-0237-y 28253908 PMC5335496

[43] Roggenbuck M, Schnell IB, Blom N, Bælum J, Bertelsen MF, Sicheritz-Pontén T, et al The microbiome of New World vultures. Nat Commun. 2014; 5: 5498 10.1038/ncomms6498 25423494

[44] Waite DW, Taylor MW. Characterizing the avian gut microbiota: membership, driving influences, and potential function. Front Microbiol. 2014; 5: 223 10.3389/fmicb.2014.00223 24904538 PMC4032936

[45] Taylor MJ, Mannan RW, U’Ren JM, Garber NP, Gallery RE, Arnold AE. Age-related variation in the oral microbiome of urban Cooper’s hawks (*Accipiter cooperii*). BMC Microbiol. 2019; 19(1): 47 10.1186/s12866-019-1413-y 30791867 PMC6385412

[46] Budachetri K, Williams J, Mukherjee N, Sellers M, Moore F, Karim S. The microbiome of neotropical ticks parasitizing on passerine migratory birds. Tick Borne Dis. 2017; 8(1): 170–173. 10.1016/j.ttbdis.2016.10.014 PMC5472101 27802919

[47] Kaltenpoth M, Steiger S. Unearthing carrion beetles' microbiome: characterization of bacterial and fungal hindgut communities across the Silphidae. Molecular ecology. 2014; 23(6): 1251–1267. 10.1111/mec.12469 24102980

[48] Grond K, Sandercock BK, Jumpponen A, Zeglin LH. The avian gut microbiota: community, physiology and function in wild birds. J Avian Biol. 2018; 49(11): e01788.

[49] Crespo R, Dowd SE, Varland DE, Ford S, Hamer TE. Bacterial Diversity in Feces of Wild Bald Eagles, Turkey Vultures and Common Ravens from the Pacific Northwest Coast, USA. BioRxiv. 2019; 511147.

[50] Dale C, Beeton M, Harbison C, Jones T, Pontes M. Isolation, pure culture, and characterization of “Candidatus *Arsenophonus arthropodicus*,” an intracellular secondary endosymbiont from the hippoboscid louse fly Pseudolynchia canariensis. Appl Environ Microbiol. 2006; 72(4): 2997–3004. 10.1128/AEM.72.4.2997-3004.2006 16598007 PMC1449044

[51] Grindle N, Tyner JJ, Clay K, Fuqua C. Identification of Arsenophonus-type bacteria from the dog tick *Dermacentor variabilis*. J Invertebr Pathol. 2003; 83: 264–266. 10.1016/s0022-2011(03)00080-6 12877836

[52] Thao ML, Baumann P. Evidence for multiple acquisition of Arsenophonus by whitefly species (Sternorrhyncha: Aleyrodidae). Curr Microbiol. 2004; 48: 140–144. 10.1007/s00284-003-4157-7 15057483

[53] Tsuchida T, Koga R, Shibao H, Matsumoto T, Fukatsu T. Diversity and geographic distribution of secondary endosymbiotic bacteria in natural populations of the pea aphid, *Acyrthosiphon pisum*. Mol Ecol. 2002; 11: 2123–2135. 10.1046/j.1365-294x.2002.01606.x 12296954

[54] Russell JA, Latorre A, Sabater-Munoz B, Moya A, Moran NA. Side-stepping secondary symbionts: widespread horizontal transfer across and beyond the Aphidoidea. Mol Ecol. 2003; 12: 1061–1075. 10.1046/j.1365-294x.2003.01780.x 12753224

[55] Subandiyah S, Nikoh N, Tsuyumu S, Somowiyarjo S, Fukatsu T. Complex endosymbiotic microbiota of the citrus psyllid *Diaphorina citri* (Homoptera: Psylloidea). Zoolog Sci. 2000; 17: 983–989.

[56] Gherna RL, Werren JH, Weisburg W, Cote R, Woese CR, Mandelco L, et al Arsenophonus nasoniae gen. nov., sp. nov., the causative agent of the son-killer trait in the parasitic wasp *Nasonia vitripennis*. Int J Syst Bacteriol. 1991; 41: 563–565.

[57] Nováková E, Hypša V, Nguyen P, Husník F, Darby AC. Genome sequence of Candidatus *Arsenophonus lipopteni*, the exclusive symbiont of a blood sucking fly *Lipoptena cervi* (Diptera: Hippoboscidae). Stand Genomic Sci. 2016; 11(1): 72. 27660670 10.1186/s40793-016-0195-1 PMC5027103

[58] Beard CB, Dotson EM, Pennington PM, Eichler S, Cordon-Rosales C, Durvasula RV. Bacterial symbiosis and paratransgenic control of vector-borne Chagas disease. Int J Parasitol. 2001; 31(5–6): 621–627. 10.1016/s0020-7519(01)00165-5 11334952

[59] Gerlach H. Bacteria Avian Medicine: Principles and Application. Wingers Publishing Inc 1994; 951–983.

[60] Junqueira ACM, Ratan A, Acerbi E, Drautz-Moses DI, Premkrishnan BN, Costea PI, et al The microbiomes of blowflies and houseflies as bacterial transmission reservoirs. Scientific reports. 2017; 7(1): 16324 10.1038/s41598-017-16353-x 29176730 PMC5701178

[61] Davidsson PR, Kariola T, Niemi O, Palva ET. Pathogenicity of and plant immunity to soft rot pectobacteria. Front Plant Sci. 2013; 4: 191 10.3389/fpls.2013.00191 23781227 PMC3678301

[62] Gurung K, Wertheim B, Falcao Salles J. The microbiome of pest insects: it is not just bacteria. Entomol Ex Appl. 2019; 167(3): 156–170.

[63] Hernández-García JA, Briones-Roblero CI, Rivera-Orduña FN, Zúñiga G. Revealing the gut bacteriome of Dendroctonus bark beetles (Curculionidae: Scolytinae): diversity, core members and co-evolutionary patterns. Scientific reports. 2017; 7(1): 13864 10.1038/s41598-017-14031-6 29066751 PMC5655642

[64] Behar A, Yuval B, Jurkevitch E. Gut bacterial communities in the Mediterranean fruit fly (*Ceratitis capitata*) and their impact on host longevity. J Insect Physiol. 2008; 54: 1377–1383. 10.1016/j.jinsphys.2008.07.011 18706909

[65] Rizzi A, Crotti E, Borruso L, Jucker C, Lupi D, Colombo M, et al Characterization of the bacterial community associated with larvae and adults of *Anoplophora chinensis* collected in Italy by culture and culture-independent methods. Biomed Res Int. 2013; 2013: 420287 10.1155/2013/420287 24069601 PMC3771249

[66] Kasana RC, Pandey CB. Exiguobacterium: an overview of a versatile genus with potential in industry and agriculture. Crit Rev Biotechnol. 2018; 38(1): 141–156. 10.1080/07388551.2017.1312273 28395514

[67] Pitt TL, Malnick H, Shah J, Chattaway MA, Keys CJ, Cooke FJ, et al Characterisation of *Exiguobacterium aurantiacum* isolates from blood cultures of six patients. Clin Microbiol Infect. 2007; 13(9): 946–948. 10.1111/j.1469-0691.2007.01779.x 17645563

[68] Ishii Y, Matsuura Y, Kakizawa S, Nikoh N, Fukatsu T. Diversity of bacterial endosymbionts associated with Macrosteles leafhoppers vectoring phytopathogenic phytoplasmas. Appl Environ Microbiol. 2013; 79(16): 5013–5022. 10.1128/AEM.01527-13 23770905 PMC3754707

[69] Sonawane MS, Chaudhary RD, Shouche YS, Sayyed RZ. (2018). Insect gut bacteria: a novel source for siderophore production. PNAS India Section B: Biol Sci. 2018; 88(2): 567–572.

[70] Kim CH, Lampman RL, Muturi EJ. Bacterial communities and midgut microbiota associated with mosquito populations from waste tires in East-Central Illinois. J Med Entomol. 2015; 52(1): 63–75. 10.1093/jme/tju011 26336281

[71] Goodrich LJ, Crocoll ST, Senner SE. Broad-winged Hawk (Buteo platypterus). In: Poole A, editor. The Birds of North American Online. Ithaca: Cornell Lab of Ornithology; 2014. Retrieved from the Birds of North American Online: http://bna.birds.cornell.edu/bna/species/218doi:10.2173/bna.218.

[72] Toth E, Kovács G, Schumann P, Kovács AL, Steiner U, Halbritter A, et al Schineria larvae gen. nov., sp. nov., isolated from the 1st and 2nd larval stages of *Wohlfahrtia magnifica* (Diptera: Sarcophagidae). Int J Syst Evol Micro. 2001; 51(2): 401–407. 10.1099/00207713-51-2-401 11321085

[73] Toth EM, Hell E, Kovács G, Borsodi AK, Marialigeti K. Bacteria isolated from the different developmental stages and larval organs of the obligate parasitic fly, *Wohlfahrtia magnifica* (Diptera: Sarcophagidae). Microb Ecol. 2006; 51(1): 13–21. 10.1007/s00248-005-0090-6 16382282

[74] Zheng L, Crippen TL, Singh B, Tarone AM, Dowd S, Yu Z, et al A survey of bacterial diversity from successive life stages of black soldier fly (Diptera: Stratiomyidae) by using 16S rDNA pyrosequencing. J Med Entomol. 2013; 50(3): 647–58. 10.1603/me12199 23802462

[75] Singh B, Crippen TL, Zheng L, Fields AT, Yu Z, Ma Q, et al A metagenomic assessment of the bacteria associated with *Lucilia sericata* and *Lucilia cuprina* (Diptera: Calliphoridae). Appl Microbiol Biotechnol. 2015; 99(2): 869–83. 10.1007/s00253-014-6115-7 25306907

[76] Levin II, Parker PG. Infection with Haemoproteus iwa affects vector movement in a hippoboscid fly—frigatebird system. Mol Ecol. 2014; 23(4): 947–53. 10.1111/mec.12587 24215498

[77] Swei A, Kwan JY. Tick microbiome and pathogen acquisition altered by host blood meal. ISME J. 2017; 11(3): 813 10.1038/ismej.2016.152 27858931 PMC5322304

[78] Coon KL, Vogel KJ, Brown MR, Strand MR. Mosquitoes rely on their gut microbiota for development. Mol Ecol. 2014; 23(11): 2727–2739. 10.1111/mec.12771 24766707 PMC4083365

[79] Dillon RJ, Dillon VM. The gut bacteria of insects: nonpathogenic interactions. Annu Rev Entomol. 2004; 49(1): 71–92. 14651457 10.1146/annurev.ento.49.061802.123416

[80] Weiss B, Aksoy S. Microbiome influences on insect host vector competence. Trends Parasitol. 2011; 27(11): 514–522. 10.1016/j.pt.2011.05.001 21697014 PMC3179784

[81] Dennison NJ, Jupatanakul N, Dimopoulos G. The mosquito microbiota influences vector competence for human pathogens. Current Opinion in Insect Science. 2014; 3: 6–13. 10.1016/j.cois.2014.07.004 25584199 PMC4288011

[82] Jupatanakul N, Sim S, Dimopoulos G. The insect microbiome modulates vector competence for arboviruses. Viruses. 2014; 6(11): 4294–4313. 10.3390/v6114294 25393895 PMC4246223

[83] Xi Z, Ramirez JL, Dimopoulos G. The *Aedes aegypti* toll pathway controls dengue virus infection. PLoS Pathogens. 2008; 4(7): e1000098 10.1371/journal.ppat.1000098 18604274 PMC2435278

[84] Narasimhan S, Fikrig E. Tick microbiome: the force within. Trends Parasitol. 2015; 31: 315–323. 10.1016/j.pt.2015.03.010 25936226 PMC4492851

[85] Narasimhan S, Rajeevan N, Liu L, Zhao YO, Heisig J, Pan J, et al Gut microbiota of the tick vector *Ixodes scapularis* modulate colonization of the Lyme disease spirochete. Cell Host Microbe. 2014; 15(1): 58–71. 10.1016/j.chom.2013.12.001 24439898 PMC3905459

[86] Guernier V, Hochberg ME, Guégan JF. Ecology drives the worldwide distribution of human diseases. PLoS Biology. 2004; 2: 740–746. 10.1371/journal.pbio.0020141 PMC423130 15208708

[87] Sehgal RN. Manifold habitat effects on the prevalence and diversity of avian blood parasites. Int J Parasitol Parasites Wildl. 2015; 4(3): 421–30. 10.1016/j.ijppaw.2015.09.001 26835250 PMC4699977

[88] Møller AP. Host–parasite interactions and vectors in the barn swallow in relation to climate change. Glob Change Biol. 2010; 16(4): 1158–1170.

[89] Descamps S. Winter temperature affects the prevalence of ticks in an Arctic seabird. PLoS One. 2013; 8(6): e65374 10.1371/journal.pone.0065374 23750259 PMC3672161

[90] Bequaert JC. The Hippoboscidae or louse-flies (Diptera) of mammals and birds. Part II. Taxonomy, evolution and revision of American genera and species. Entomol AM-NY. 1954; 34: 1–232.

