# Supplementary material for: Characterizing the microbiome of ectoparasitic louse flies feeding on migratory raptors
Source: PLoS One. 2020 Jun 4;15(6):e0234050. doi: 10.1371/journal.pone.0234050 (PMC7271990; doi:10.1371/journal.pone.0234050)
Supplement: S1 Table — Age categories of raptor species included: 1) HY = Hatch Year, 2) SY = Second Year, 3) ASY = After Second Year, and 4) PB2 or PB3 = Prebasic Molt. Sex categories of raptor species included: 1) F = Females, 2) M = Males, and 3) U = Unknown. Abbreviations for raptor species are as follows: Bald Eagle (BAEA), Broad-winged Hawk (BWHA), Cooper’s Hawk (COHA), Northern Goshawk (NOGO), Red-shouldered Hawk (RSHA), Red-tailed Hawk (RTHA), and Sharp-shinned Hawk (SSHA). (DOCX) [file pone.0234050.s001.docx]

**S1 Table. Raptor host species that louse flies were collected off of in 2015–2016 from a migration banding station in Pennsylvania, United States.** Age categories of raptor species included: 1) HY = Hatch Year, 2) SY = Second Year, 3) ASY = After Second Year, and 4) PB2 or PB3 = Prebasic Molt. Sex categories of raptor species included: 1) F = Females, 2) M = Males, and 3) U = Unknown. Abbreviations for raptor species are as follows: Bald Eagle (BAEA), Broad-winged Hawk (BWHA), Cooper’s Hawk (COHA), Northern Goshawk (NOGO), Red-shouldered Hawk (RSHA), Red-tailed Hawk (RTHA), and Sharp-shinned Hawk (SSHA).

| **Date** | **Time** | **Species of Bird** | **Age** | **Sex** | **Weight (g)** | **# Flies Collected** | |
| --- | --- | --- | --- | --- | --- | --- | --- |
| 9/8/15 | 1143 | SSHA | HY | F | 164 | 1 |  |
| 9/8/15 | 1330 | RTHA | HY | U | 1256 | 1 |  |
| 9/14/15 | 1038 | COHA | ASY | F | 480 | 1 |  |
| 9/15/15 | 1510 | SSHA | HY | F | 160 | 1 |  |
| 9/17/15 | 1500 | BAEA | PB2 or PB3 | U | – | 1 |  |
| 9/18/15 | 1020 | BWHA | HY | U | 412 | 1 |  |
| 9/18/15 | 1350 | COHA | HY | F | 504 | 2 |  |
| 9/19/15 | 1604 | SSHA | HY | F | 172 | 1 |  |
| 9/22/15 | 1333 | SSHA | HY | F | 186 | 1 |  |
| 9/24/15 | 1125 | COHA | HY | F | 540 | 2 |  |
| 9/24/15 | 1300 | RTHA | HY | U | 1204 | 7 |  |
| 9/25/15 | 1407 | SSHA | HY | M | 98 | 1 |  |
| 9/25/15 | 1241 | SSHA | HY | F | 174 | 2 |  |
| 9/26/15 | 1231 | SSHA | HY | M | 100 | 1 |  |
| 9/28/15 | 955 | SSHA | HY | M | 102 | 1 |  |
| 10/1/15 | 1105 | COHA | HY | F | 538 | 4 |  |
| 10/15/15 | 1345 | COHA | HY | F | 523 | 1 |  |
| 10/15/15 | 1100 | COHA | ASY | F | 535 | 2 |  |
| 10/16/15 | 1402 | SSHA | SY | M | 104 | 1 |  |
| 10/23/15 | 1044 | COHA | HY | M | 348 | 1 |  |
| 10/23/15 | 1040 | COHA | SY | F | 556 | 2 |  |
| 10/29/15 | 1630 | NOHA | SY | M | 341 | 1 |  |
| 11/23/15 | 1355 | RTHA | HY | U | 956 | 2 |  |
| 9/13/16 | 1050 | SSHA | HY | F | 150 | 1 |  |
| 9/13/16 | 1145 | SSHA | HY | F | 208 | 2 |  |
| 9/14/16 | 1345 | RTHA | HY | U | 866 | 3 |  |
| 9/16/16 | 1150 | RTHA | HY | U | 1156 | 1 |  |
| 9/16/16 | 1135 | SSHA | HY | F | 178 | 2 |  |
| 9/16/16 | 1510 | RTHA | SY | U | 906 | 2 |  |
| 9/16/16 | 915 | BWHA | ASY | U | 492 | 3 |  |
| 9/16/16 | 1330 | RTHA | HY | U | 1156 | 4 |  |
| 9/16/16 | 1050 | RTHA | HY | U | 944 | 6 |  |
| 9/21/16 | 1541 | BWHA | HY | U | 561 | 1 |  |
| 9/22/16 | 1148 | RTHA | HY | U | 1107 | 2 |  |
| 9/22/16 | 1205 | RTHA | HY | U | 1134 | 2 |  |
| 9/22/16 | 1115 | RTHA | HY | U | 993 | 2 |  |
| 9/22/16 | 1300 | RTHA | HY | U | 973 | 2 |  |
| 9/23/16 | 935 | BWHA | HY | U | 403 | 1 |  |
| 9/26/16 | 1150 | SSHA | HY | F | 152 | 1 |  |
| 9/26/16 | 1405 | RTHA | HY | U | 926 | 1 |  |
| 9/26/16 | 1055 | RTHA | HY | U | 993 | 2 |  |
| 10/4/16 | 1416 | SSHA | HY | M | 104 | 1 |  |
| 10/4/16 | 1400 | SSHA | HY | M | 120 | 1 |  |
| 10/4/16 | 1500 | RTHA | HY | U | 1022 | 2 |  |
| 10/4/16 | 1330 | RTHA | HY | U | 954 | 4 |  |
| 10/6/16 | 1300 | RTHA | HY | U | 1269 | 2 |  |
| 10/7/16 | 1150 | RTHA | HY | U | 1078 | 1 |  |
| 10/9/16 | 843 | RTHA | HY | U | 1036 | 1 |  |
| 10/15/16 | 1350 | RTHA | HY | U | 762 | 1 |  |
| 11/1/16 | 1130 | RSHA | HY | U | 632 | 1 |  |
| 11/1/16 | 1400 | RTHA | HY | U | 809 | 1 |  |
| 11/1/16 | 1105 | RTHA | HY | U | 1266 | 1 |  |
| 11/1/16 | 1140 | RTHA | HY | U | 894 | 1 |  |
| 11/1/16 | 1240 | RTHA | HY | U | 976 | 1 |  |
| 11/1/16 | 1300 | RTHA | HY | U | 940 | 1 |  |
| 11/1/16 | 1418 | COHA | HY | M | 408 | 1 |  |
| 11/1/16 | 1118 | RSHA | HY | U | 588 | 2 |  |
| 11/1/16 | 1050 | RTHA | HY | U | 1288 | 3 |  |
| 11/1/16 | 1505 | RTHA | HY | U | 1245 | 6 |  |
| 11/2/16 | 957 | RTHA | HY | U | 1127 | 1 |  |
| 11/4/16 | 1045 | RTHA | HY | U | 866 | 1 |  |
| 11/4/16 | 1118 | RTHA | ASY | U | 940 | 1 |  |
| 11/7/16 | 1350 | SSHA | HY | F | 164 | 1 |  |
| 11/7/16 | 1027 | RTHA | HY | U | 1354 | 1 |  |
| 11/7/16 | 1049 | RTHA | HY | U | – | 1 |  |
| 11/10/16 | 1203 | RTHA | HY | U | 923 | 1 |  |
| 11/11/16 | 1055 | NOGO | HY | M | 830 | 1 |  |
| 11/11/16 | 1215 | SSHA | SY | F | 186 | 2 |  |
| 11/11/16 | 1245 | RTHA | HY | U | 1170 | 2 |  |
| 11/12/16 | 1145 | NOGO | HY | M | 804 | 3 |  |
| 11/26/16 | 1159 | RTHA | HY | U | 944 | 1 |  |
